# Diagnosis and Progression of Sacroiliitis in Repeated Sacroiliac Joint Computed Tomography

**DOI:** 10.1155/2013/659487

**Published:** 2013-09-03

**Authors:** Mats Geijer, Gro Gadeholt Göthlin, Jan H. Göthlin

**Affiliations:** ^1^Center for Medical Imaging and Physiology, Skåne University Hospital, Lund University, 221 85 Lund, Sweden; ^2^Department of Diagnostic Radiology, Sahlgrenska University Hospital, University of Gothenburg, 413 45 Gothenburg, Sweden; ^3^Department of Diagnostic Radiology, Sahlgrenska University Hospital, University of Gothenburg, 431 30 Mölndal, Sweden

## Abstract

*Objective*. To assess the clinical utility of repeat sacroiliac joint computed tomography (CT) in sacroiliitis by assessing the proportion of patients changing from normal to pathologic at CT and to which degree there is progression of established sacroiliitis at repeat CT. *Methods*. In a retrospective analysis of 334 patients (median age 34 years) with symptoms suggestive of inflammatory back pain, CT had been performed twice, in 47 of these thrice, and in eight patients four times. The studies were scored as normal, equivocal, unilateral sacroiliitis, or bilateral sacroiliitis. *Results*. There was no change in 331 of 389 repeat examinations. Ten patients (3.0%) had progressed from normal or equivocal to unilateral or bilateral sacroiliitis. Of 43 cases with sacroiliitis on the first study, 36 (83.7%) progressed markedly. Two normal cases had changed to equivocal. Eight equivocal cases were classified as normal on the repeat study. In further two patients, only small changes within the scoring grade equivocal were detected. *Conclusions*. CT is a valuable examination for diagnosis of sacroiliitis, but a repeated examination detects only a few additional cases of sacroiliitis. Most cases with already established sacroiliitis showed progression of disease.

## 1. Introduction

Radiology plays an important role in diagnostic criteria for ankylosing spondylitis (AS) such as the New York (NY) criteria [1] and the modified NY criteria [2]. In classification criteria of spondyloarthropathies, it has been of minor importance [3, 4]. However, the recent Assessment of SpondyloArthritis International Society (ASAS) criteria from 2009 require sacroiliitis on imaging plus one or more spondyloarthritis features or positive serology for HLA-B27 plus two or more spondyloarthritis features for spondyloarthropathy classification [5]. 

Computed tomography (CT) has notably higher accuracy than radiography in imaging of the sacroiliac joints [6], especially for evaluation of chronic changes from sacroiliitis. Even though both sensitivity and specificity of CT are high, some examinations cannot with certainty be classified as either normal (i.e., showing no or only incidental degenerative or other findings) or as showing unequivocal sacroiliitis, as is the case with all imaging modalities. 

A review of the literature reveals the usually long delay between onset of clinical symptoms and definite radiographic signs of sacroiliitis [7–9]. It has, however, been reported that patients with spondyloarthropathy may show significantly increased inflammatory joint changes already at one-year follow-up, with CT as well as with magnetic resonance imaging (MRI) [10].

The purpose of the current study on a clinical material was to assess the clinical utility of repeat CT in the evaluation of suspected sacroiliitis by evaluating to which extent normal or equivocal examinations may progress into manifest unequivocal sacroiliitis and to which extent there is progression of manifest sacroiliitis at repeat CT.

## 2. Material and Methods

In a retrospective review, 723 CT examinations on 334 consecutive patients between 1981 and 2011 at the Sahlgrenska University Hospital were identified in the radiology archive and the radiology information system (RIS). Inclusion criteria were (a) referral to CT because of possible inflammatory sacroiliitis, (b) patient age over 18 years, (c) more than one CT performed, and (d) images available for review. For 304 patients, at least one of the referrals for CT had come from a rheumatologist. For the remaining 30 patients, the referrals had come from orthopedic surgeons or general practitioners.

In 220 women (age range 18–72 years) and 114 men (age range 19–70 years) median age at the first CT examination was 34 years, and mean was age 35 years. The mean time between examinations one and two was four years (range < 1–21 years) for 334 patients. In 47 patients, a third examination had been made (mean time between examinations one and three was eight years, range 1–22 years) and in eight patients also a fourth CT examination (mean time between examinations one and four was 13 years, range 8–22 years).

Before 2006, studies had been done using single-slice CT scanners with a high resolution algorithm and maximum gantry tilt to obtain oblique coronal images. As the gantry tilt ability was limited, the examinations were performed with the patient prone with a pillow under the hips. Studies using more modern CT scanners with 30 degrees gantry tilt ability were performed with the patient supine. The images were 3 mm thick in most cases, always contiguous, and covered the entire synovial part of the sacroiliac joints. After 2006, all studies were done using multidetector imaging, with oblique coronal reconstructions. Oblique coronal sectional CT images, parallel to the anterior border of the sacrum, were available for review in all patients.

All images were reviewed and analyzed for radiologic signs of sacroiliitis by two investigators with previously reported good interobserver agreement [11], blinded to the clinical data and original reports, but not blinded to name and age on the films. Studies before 2003 were read as hard copies, later studies as soft copies. Each joint on each study was scored separately. A direct comparison of the studies was allowed for the second, third, and fourth examinations. The sacroiliac joints were graded as normal (with or without degenerative changes), equivocal for sacroiliitis, or as showing unilateral or bilateral sacroiliitis according to the NY criteria [1]. The classification was thus a modification of the NY criteria, where radiography had been replaced by CT. The films were reviewed in chronological order and a side-by-side comparison of the examinations in each patient was done to evaluate progression or regression of changes. The follow-up examination was scored as unchanged, as showing minor changes within the same NY criteria scoring grade, as showing development of sacroiliitis from a previously normal or equivocal examination, or as showing progression of sacroiliitis in already established disease. In cases with disagreement, consensus was reached among all three authors at a second review.

## 3. Results

At the first examination, 43 of 334 CT examinations showed sacroiliitis, 21 of these bilateral and 22 unilateral (Table 1), with all patients having AS according to the NY criteria. Sixty-one examinations were equivocal and 230 normal. 

There were totally 389 follow-up examinations on 334 patients. Of these examinations, 331 (85.1%) were unchanged between CT examinations. In 282 of the 334 patients (84.4%) there was no change in grading of CT examinations between examination one and two (Table 2). Median patient age was 34 years at the first examination, range 18–66 years. Median interval from the first CT examination was three years, range 0–21 years. 

**Figure 1:**
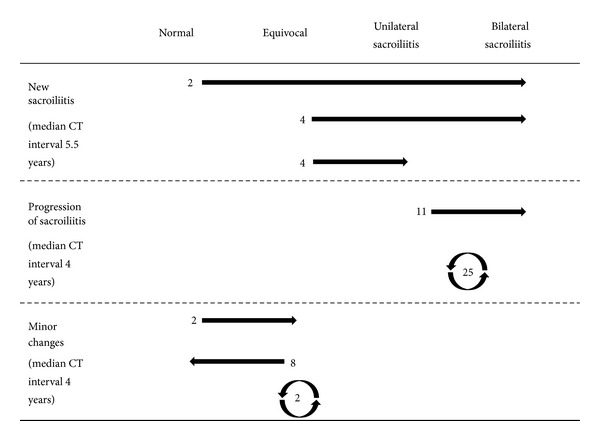
Changes in sacroiliitis grading in 58 of 334 patients with CT of the sacroiliac joints. In 25 cases with sacroiliitis and two equivocal cases, the observed changes were too small to merit a change of sacroiliitis grade.

**Figure 2:**
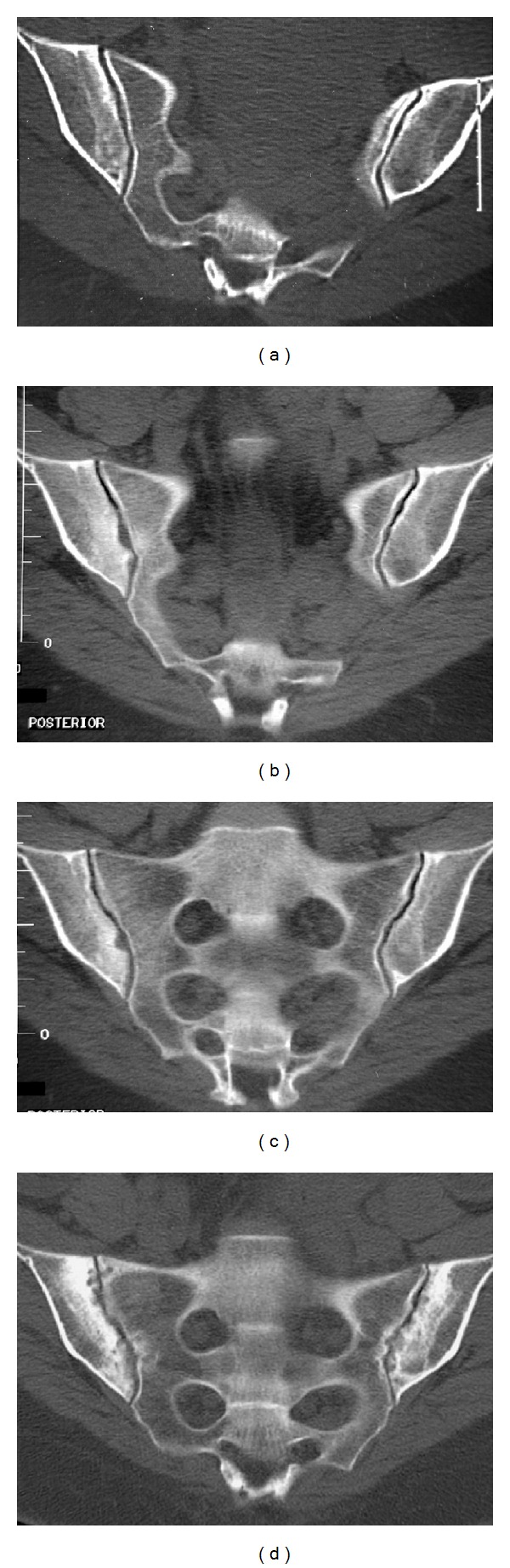
A female patient with three studies showing progression from equivocal to unilateral to advanced bilateral sacroiliitis. (a) The initial equivocal CT, obtained at age 26, shows mild asymmetric sclerosis on the iliac side of the right sacroiliac joint, with a suggestion of a single erosion and slightly poor definition of the iliac subchondral cortex. (b, c) After two years, the suspected changes have progressed into definite unilateral sacroiliitis, with broad erosions and dense inflammatory sclerosis surrounding the right sacroiliac joint. (d) After an additional 11 years, the disease has progressed into bilateral advanced sacroiliitis, with widespread erosions and inflammatory sclerosis affecting both the sacral and iliac sides of the joints.

**Figure 3:**
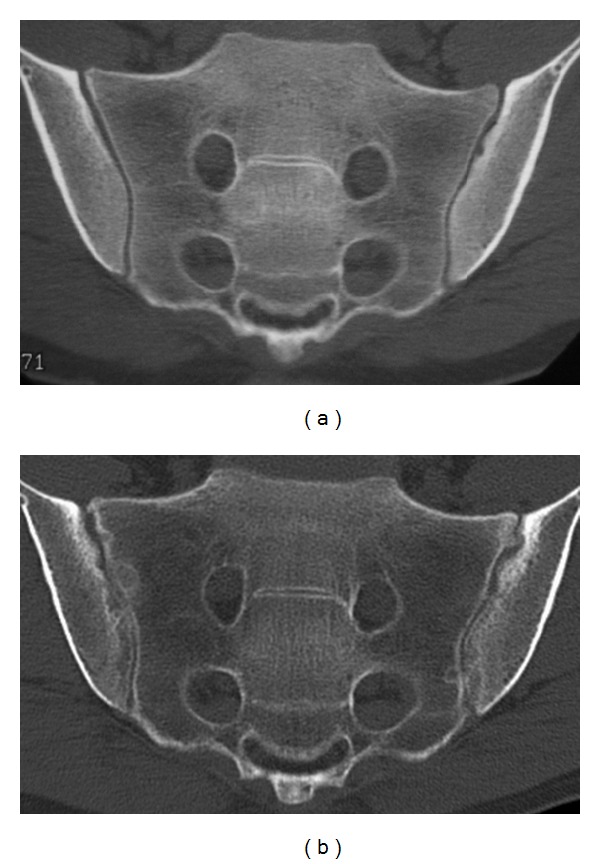
A case with changes from unilateral to bilateral sacroiliitis in a 25-year-old male with 1–3 years history of suspected spondyloarthropathy. (a) The initial CT shows unilateral sacroiliitis with subtle erosions in the left sacroiliac joint on the iliac side, with surrounding mild inflammatory sclerosis. On the right, there is poor definition of the subchondral cortical bone. (b) Seven years later, there is progression to definite bilateral sacroiliitis.

**Figure 4:**
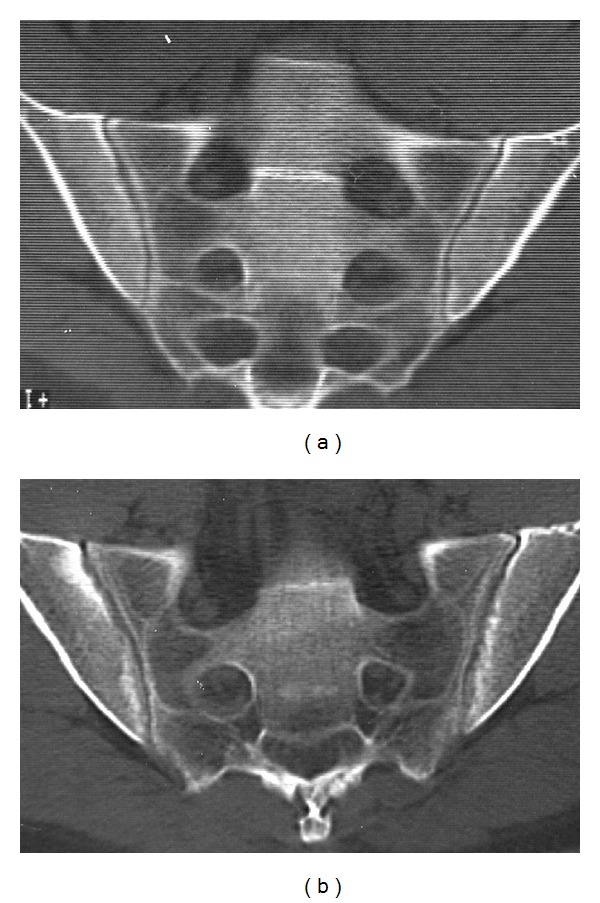
A 37-year-old male with 1–3 years duration of inflammatory low back pain. (a) The initial CT shows unilateral sacroiliitis with subtle but definite erosions on the iliac side of the right sacroiliac joint. (b) After 5.5 years, there is progression to bilateral moderately advanced sacroiliitis.

Totally, 58 of the 389 follow-up examinations had changed sacroiliitis grading from the first examination (Figure 1). There were 10 new cases of sacroiliitis: Two normal examinations and four equivocal examinations had advanced to bilateral sacroiliitis, and four equivocal examinations to unilateral sacroiliitis (Figure 2). Median examination interval for these 10 patients (3.0% of all patients) with onset of CT findings or progression of disease from equivocal to manifest sacroiliitis between CT examination one and two (in one case examination three) was 5.5 years, with range 1–21 years. There were 36 cases with progression of sacroiliitis. Eleven examinations had advanced from unilateral to bilateral sacroiliitis (Figures 2, 3, and 4), and 25 examinations with unilateral or bilateral changes showed progressively more advanced sacroiliitis. Median examination interval for these 36 patients with progression of already established sacroiliitis between CT examination one and two (in two cases examination three) was four years, with range 0–17 years. In the remaining 12 examinations with minor changes without change of NY criteria scoring grade, two normal examinations had advanced to equivocal, eight equivocal examinations had reverted to normal, and two examinations with equivocal changes had advancing changes without turning into definite sacroiliitis. For these patients, median examination interval was 4 years, with range 0–17 years.

Patients presenting with new sacroiliitis were of about the same median age as the entire study population, while patients with progression of sacroiliitis were slightly younger than the rest (Table 2).

## 4. Discussion

In the current study on the value of repeat CT of the sacroiliac joints to diagnose chronic destructive or reparative changes after sacroiliitis, an effort was made to answer the question whether repeat CT will detect additional cases of sacroiliitis after the first CT and, if so, at what frequency. At our institution, sacroiliac joint CT is common, and repeat CT is not uncommon under the presumption that additional cases of sacroiliitis will slowly manifest themselves over time. We have also tried to evaluate to which degree changes from established sacroiliitis show progression over time.

The value of CT as a diagnostic tool in diagnosing sacroiliitis in suspected spondyloarthritis has been shown in several previous reports [6, 12–19]. All CT examinations in the current study were performed before treatment with biologic agents for spondyloarthritis was approved.

To our knowledge, this is the first study to evaluate repeat CT for this indication, and neither has to our knowledge such a study has been published on the diagnostic value of repeated MRI in an unselected material. Published reports either address changes over time in carefully selected patients with clinical sacroiliitis [10] or use MRI to monitor treatment [20]. The use of repeated radiographic examinations to diagnose radiographic sacroiliitis before MRI became available was common. A large number of studies have included sequential radiographic imaging in the evaluation of disease progression [21]. The slow progression in a few cases in the current study from normal or equivocal, and the progression of sacroiliitis in established disease, is similar to the slow rate of progression of radiographic changes found in those studies [22].

In an earlier report, 25.3% pathologic outcome in primary clinical CT for suspected sacroiliitis was demonstrated [11]. In the present study, the repeat CT examinations yielded few new cases (*n* = 10, 3.0%) where patients with initially normal or equivocal examinations on repeat examination had showed manifest destructive or reparative changes after sacroiliitis. The results in the current study confirm that primary CT for diagnosis of sacroiliitis has a high diagnostic value, where few cases are missed or progress from normal or equivocal into destructive or reparative changes after sacroiliitis, but also show that repeat CT has a clearly lower diagnostic value compared with primary CT. In cases with negative CT and remaining high clinical suspicion of sacroiliitis, it would seem more reasonable to perform MRI as a secondary investigation instead of repeating the CT. Due to lacking clinical data in the current study about symptom duration, it is impossible to draw any conclusion about how soon after onset of symptoms a CT examination might be possible. In a study on 1304 patients with sacroiliac joint CT [14] from our institution, about half of the patients with clinical data had had symptoms for less than six years. However, adequate clinical data were lacking in almost half of the patients. It has previously been reported that changes in CT appearance and scoring are possible after a follow-up period of only one year in a study group of patients with early spondyloarthropathy [10]. The results from that study cannot however be directly compared to the current study, since there are large differences in the patient selection and in the scoring methods. Most patients with established destructive or reparative changes after sacroiliitis at the first examination in the current study did progress over a mean time of 5.5 years.

In the current study, 10 repeat CT examinations were downgraded from equivocal to normal. In these examinations, suspected erosion-like changes in the joint surface could not be reproduced at the follow-up CT, perhaps due to partial volume effect and slight differences in slice angulation between the examinations. Similarly, there were a number of examinations with slight variation of appearance from the previous examination but within the same NY scoring grade. This variation in interpretation and misinterpretation results in observer variance, which has previously reported in a large study population [11]. It has also been reported that spontaneous regression of radiographic changes may occur in reactive arthritis [23]. 

In CT of the sacroiliac joints, optimal image quality and viewing conditions are important to detect early and subtle changes [19]. The multiplanar reconstruction (MPR) images should not be thicker than 3 mm, and the CT examinations should be read on a picture archiving and communication system (PACS) with MPR capability. Some of the patients in the current study with obvious progression of destructive or reparative changes after sacroiliitis from the first to the second examination had subtle changes on the first examination such as minimal erosions or subtle subchondral sclerosis (Figures 2, 3, and 4) which were visible in retrospect but may be overlooked when reading the CT studies, especially if the window and level settings do not allow for good review of the subchondral cortex and trabecular bone. Bone marrow edema is the first diagnostic sign to appear, visible on MRI but hard to detect on CT. Bone marrow edema can however be visualized at CT as well as with MRI under certain conditions [24, 25]. CT diagnosis is based on destructive or reparative changes such as sclerosis or erosions, and erosions have been reported as the most definite sign of sacroiliitis in CT [14] as well as in MRI [26]. Knowledge about the CT appearance of sacroiliitis is important to avoid overdiagnosis [27].

Already in 1981, sacroiliac joint CT was shown to have a higher sensitivity and specificity for sacroiliitis than radiography [28, 29]. CT was rapidly implemented at our institution and was quickly accepted by the rheumatologists, why only a minority of patients also had sacroiliac joint radiography performed. MRI became available at our institution in 1991, but clinical sacroiliac joint MRI only gained popularity a few years after 2000, and thus, only few patients in the current study also have had an MRI study of the sacroiliac joints.

Sacroiliitis is nearly impossible to detect with CT before structural changes have appeared, which is a limitation compared with MRI. In addition, radiography, CT, and bone scintigraphy all give a radiation dose to the patient. In a study from 2002, it was reported that the effective dose from a semicoronal CT of the sacroiliac joints is 100 *μ*Sv for men and 102 *μ*Sv for women, compared to anterior-posterior (AP) projection radiography of the sacroiliac joints resulting in 39 *μ*Sv for men and 255 *μ*Sv for women [30]. Dose levels for sacroiliac joint CT from modern multidetector CT scanners with modern dose reducing image reconstruction algorithms are not available. A bone scan results in an effective dose of about 4 mSv. In comparison, the background radiation is about 2.4 mSv per year in the world, with large variations between countries.

There are limitations to the current study. It is retrospective, where the quality of the clinical information varied, since the information was obtained from the referral forms. This also means that there has been no clinical diagnostic correlation and there is no uniform information about which patients had been diagnosed with AS or another of the spondyloarthropathies or which patients eventually received a diagnosis of no spondyloarthritis. All patients had however been referred to CT for clinical suspicion of sacroiliitis, with a clear majority of the patients referred by rheumatologists, and the likelihood of spondylarthritis in the patient group as a whole had probably been seen as high by the referrers, since examinations done before 1997 had been part of a previous report on CT of the sacroiliac joints [11] in which 25.3% showed uni- or bilateral sacroiliitis. The CT examinations in the current report had been done over a period of several years where the earlier studies sometimes had a slightly lower quality than the more recent. A direct comparison of the studies was allowed for the second, third, and fourth examinations. There are pros and cons to such a nonrandom scoring order [31], with a certain amount of expectation bias being introduced and possible overestimation of change, such as reported for scoring spinal radiographs in ankylosing spondylitis [32], where a paired scoring (the same patient's images in unknown chronological order) was preferred. On the other hand, a paired reading order may underestimate progression [33].

In conclusion, the clinical utility of CT is good in establishing a diagnosis of sacroiliitis, but repeat CT has a low utility. CT detected development from initially normal to later sacroiliitis in only 10 of 334 cases. Most cases (28 of 35) with established sacroiliitis showed marked progression of disease. 

## Figures and Tables

**Table 1:** Number of patients with normal, equivocal, or pathologic findings at initial and repeat CT, in 334 patients with suspected sacroiliitis.

Examination number	Normal	Equivocal	Unilateral sacroiliitis	Bilateral sacroiliitis	Total
1	230 (68.9%)	61 (18.3%)	22 (6.6%)	21 (6.3%)	334 (100%)
2	223 (66.8%)	60 (18.0%)	14 (4.2%)	37 (11.1%)	334 (100%)
3	38	5	1	3	47
4	6	1		1	8

**Table 2:** Summary of patients' ages at examination two and interval between examinations one and two for patients with no interval changes, minor CT changes within the same scoring grade, progression into sacroiliitis, and progression of already established sacroiliitis. Age and examination interval in years.

	No changes between examinations	New sacroiliitis	Progression of sacroiliitis	Minor changes	All patients
Number	282	9	34	9	334
Median patient age	38	39	34.5	32	38
Patient age range	19–72	29–58	21–66	27–45	19–72
Median interval between examinations	3	5	4	3	4
Range of interval between examinations	<1–21	1–21	<1–16	<1–17	<1–21
